# Uses of 2-Ethoxy-4(3*H*) quinazolinone in Synthesis of Quinazoline and Quinazolinone Derivatives of Antimicrobial Activity: The Solvent Effect

**DOI:** 10.5539/gjhs.v4n1p162

**Published:** 2012-01-01

**Authors:** Maher A. El-Hashash, Sameh A. Rizk, Fakhry A. El-Bassiouny, Khalid M. Darwish

**Affiliations:** Chemistry Department, Faculty of Science, Ain Shams University Abbassia, Cairo, Egypt; Chemistry Department, Science Faculty, University of Garyounis, Benghazi, Libya Tel: 002-011-3049976 E-mail: khaliddarwish1962@yahoo.com

**Keywords:** Quinazolinone, Quinazoline, Tautomerisation, Nucleosides, Chalcone

## Abstract

2- Ethoxy-4(3*H*) quinazolinone 1 was synthesized and allowed to react with various halides, namely: alkyl, benzyl, allyl, acyl, haloacetyl, crotonyl, benzoyl, 2-furoyl and 1-naphthalenesulphonyl halides affording quinazoline and quinazolinone derivatives. The reactions of compound 1 with phosphorus oxychloride, phosphorus pentasulfide, ethyl chloroformate, ethyl chloroacetate, α-bromoglucose tetraacetate, *p*-acylaminobenzenesulfonyl chloride, acrylonitrile, chalcone and chalcone oxide were also investigated. Depending on the reaction condition and reactant halide, the type of substituent (alkyl, acyl, aroyl, etc.) that will reside on either of the expected positions (3 or 4) on the quinazoline moiety can control the reaction pathway for synthesis of the promising products. The significant role of solvent responsible for determining both the reaction pathway and type of products synthesized was also discussed. Some derivatives were chosen for biological screening test against Gram (-ive) and Gram (+ive) bacteria and two strains of fungi.

## 1. Introduction

In recent years there has been an increasing interest in the chemistry of 4(3*H*)-quinazolinones because of their biological importance. Many of them show antifungal, antibacterial, anticancer, anti-inflammatory, anticonvulsant, immunotropic, hypolipidemic, antitumor, antiulcer, analgesic, antiproliferative activities and inhibitory effects for thymidylate synthase and poly (ADP-ribose) polymerase (PARP) [1-13]. The 4(3*H*)-quinazolinones can act as semicyclic amides or iminols, due to the tautomeric phenomenon they have. Their reactions in either form with alkyl or acyl halides are perhaps the most interesting due to the large number of heterocycles that are obtained either directly or through further transformations of the initially formed products.

## 2. Results and Disscussion

**Scheme 1:**
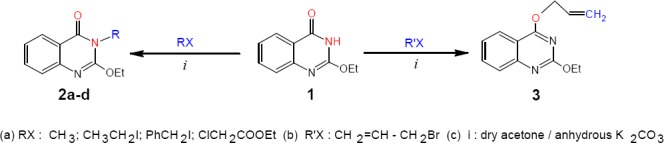
synthetic pathway for compounds **2** and **3**

By the solvent free reaction of 2-ethoxy (4*H*)-3, 1-benzoxazin-4-one with ammonium acetate 2-ethoxy (4*H*)-3,1-quinazolin-4-one 1 was synthesized to be used as the starting material. In this paper we report its behavior towards various types of organic halides, with the aim of obtaining more precise information about the course of reaction and bearing into consideration the effects of changing the reaction conditions (dry solvent, solvent free, base, etc.) on the reaction pathway to obtain the promising products. Therefore the interaction of compound 1 with alkyl halides such as methyl iodide, ethyl iodide, benzyl chloride and ethyl chloroacetate, carried out in dry acetone and anhydrous potassium carbonate gave the corresponding *N*-substituted quinazolinones 2a-d (Scheme 1), respectively. The IR spectra of products 2a-d showed absorption bands in the range 1672-1791 attributable to υ_max_ of C=O groups. But when the reaction was carried out using allyl bromide the *O*-substituted quinazoline 3 was obtained, confirming the allylic effect caused by the unsaturated moiety adjacent to the carbon atom bearing the halide. In addition, the IR spectrum of product 3 showed an absorption band at 1264 attributable to υ_max_ of (C-O-C) and no any band for the C=O group.

**Scheme 2:**
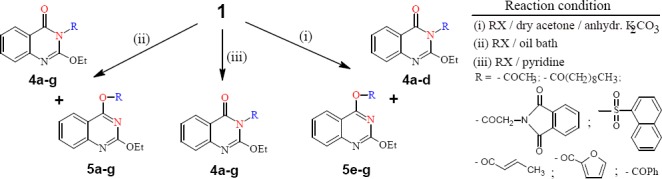
synthetic pathway for compounds **4** and **5**

Compound 1 was reacted with acid chlorides, namely: acetyl, *n*-decanoyl, phthalimidoacetyl and 1-naphthalene sulfonyl chlorides in dry acetone / anhydrous K_2_CO_3_ affording the 3-substituted 4a-d products, respectively, and with *α,β*-unsaturated or aromatic halides namely: crotonyl, furoyl and benzoyl chlorides affording the *O*-substituted derivatives 5e-g respectively. But when the reactions were done using oil bath, both the *O*-substituted 5a-g and *N*-substituted 4a-g, with lower yields of the latter. The IR spectra for 4a-d showed two groups of absorption bands in the ranges 1671-1691 and 1714-1738, which are attributable to the endocyclic and exocyclic carbonyl groups of quinazolinone derivatives, respectively, whereas the IR spectra of 5e-g showed absorption bands in the range 1763-1769 attributable to C=O groups of the ester. This can be a good proof in explaining why compound 1 when reacted, in dry acetone, with acid chlorides having the carbonyl groups directly linked to an aliphatic moiety (e. g. acetyl, decanoyl, phthalimidoacetyl) the amide form of quinazolinone enhanced the formation of the *N*-substituted quinazolinones, whereas its reactions with the *α, β*-unsaturated or aromatic acid chlorides (e. g. crotonyl, furoyl and benzoyl chlorides) allowed the imidol form to predominate enhancing the formation of *O*- substituted quinazolines. Moreover, this behavior was opposed under solvent free condition. However, in dry pyridine only products 4a-g were obtained with the formation of the more favorable pyridinium chloride. This confirmed that the more basic pyridine, compared with the only polar acetone, preferred to interact with the slightly more acidic amide form affording the pyridinium salt according to the expected reaction pathway, and therefore enhancing the formation of the 3-substituted quinazolinones, independent of the type of acid chloride used (Scheme 2). The structures of 4a-g and 5a-g were based on the microanalytical and spectral data.

**Scheme 3:**
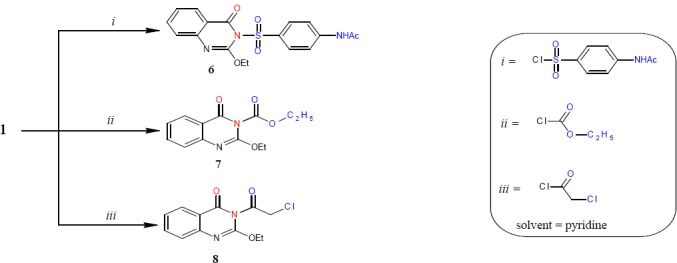
synthetic pathway for compounds **6 - 8**

Compound 1 was reacted with *p*-acetylaminobenzenesulfonyl chloride, ethyl chloroformate and chloro-acetyl chloride in dry pyridine affording derivatives 6, 7 and 8 respectively (Scheme 3). The IR spectra of products 6-8 showed two types absorption bands in the ranges 1666-1672 and 1712-1762 attributable to υ_max_ of the endocyclic C=O group of quinazolinone and exocyclic C=O groups of acetyl- aminophenyl, ethoxycarbonyl and chloroacetyl moieties, respectively.

**Scheme 4:**
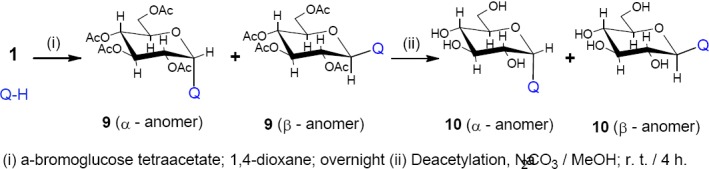
synthetic pathway for compounds **9** and **10**

It is well known that the *N*-glycosylated heterocycles are of important significance because of the cytotoxic activitiy of their acetylated derivatives, which increases dramatically after hydrolysis [14]. Herein we discuss the interaction of derivative 1 with α-bromoglucose tetraacetate in 1,4-dioxane to afford α and β anomers of the *N*-substituted acetylated quinazolinone derivative 9 followed by the deacetylation using potassium carbonate /methanol to give α and β anomers of the deacetylated product 10 (Scheme 4). The IR spectra for 9 showed two absorption bands at 1662 and 1738 attributable to υ_max_ of the endocyclic C=O group of quinazolinone and the acetyl groups of per-*O*-acetylated glucose moiety respectively. The H NMR showed no indication for the N-H of quinazolinone and elemental analysis showed no indication for the bromide atom which was originally attached to the glucose moiety. This confirmed its removal during the reaction of glucosamine with quinazolinone.

**Scheme 5:**
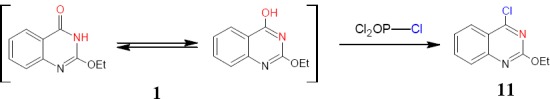
synthetic pathway for compound **11**

Moreover, compound 1, in its imidolic form, was reacted with POCl_3_/2 h affording quinazoline 11 (Scheme 5) [15]. The IR data showed no indication for the NH of quinazolinone.

**Scheme 6:**
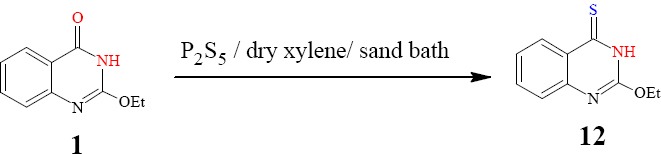
synthetic pathway for compound **12**

Compound 1 interacted with P_2_S_5_/dry xylene / sand bath / 6 h affording quinazolin-4-thione 12 (Scheme 6) [16]. The IR spectrum, in addition to the elemental analysis, showed an absorption band at 1319 attributable to υ_max_ of C=S with no indication for the C=O group.

**Scheme 7:**
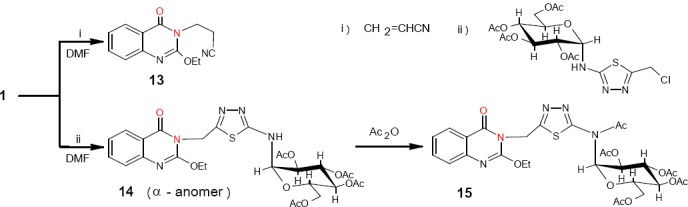
synthetic pathway for compounds **13-15**

During our attempts, it was noted that most of quinazolinones resemble secondary amines in their Michael-type addition to acrylnitrile [17]. Thus, compound 1 was treated with acrylonitrile in DMF for 5 h giving product 13 (Scheme 7). The IR spectrum showed an absorption band at 2246 attributable to υ_max_ of the CN group and the H NMR spectrum showed a triplet at δ 4.15 for the N-CH_2_ bonding.

On the other hand, compound 1 was reacted with an alkyl halide having an *N*-glucosidated thiadiazole moiety, the 2-chloromethyl-5-glucopyranosylaminothiadiazole, in DMF for 8 h affording compounds 14. The H NMR spectrum of 14 showed a singlet at δ 5.29 for the N-CH_2_ bonding. Acetylation of 14 afforded derivative 15. The IR spectrum was devoid any NH group band. Derivatives like 14 and 15 are believed to have biological activities similar to those of cyclic and acyclic nucleosides [18].

**Scheme 8:**
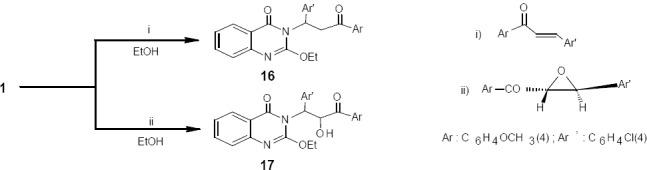
synthetic pathway for compounds **16** and **17**

Similarly, compound 1 was submitted to react with a selected chalcone and its epoxide in absolute ethanol for 5-6 h to give derivatives 16 and 17 respectively (Scheme 8). The IR spectra for 16 and 17 showed two absorption bands at 1671 and 1713-1719 characteristic for the endocyclic and exocyclic C=O groups. The H NMR spectra of 16 and 17 showed no band for the NH group of quinazolinone. Derivatives 16 and 17 are also expected to have biological activity [19].

## 3. Antimicrobial Evaluation

Compounds 4b, 4c, 4d, 4f, 4g, 6, 7, 8, 9, 10 and 15 were tested for antimicrobial activity against *Escherichia coli* (Gram negative bacterium), *Staphylococcu*s *aureus* (Gram positive bacterium), *Aspergillus flavus* and *Candida albicans* (fungi) using the disc diffusion method. The antimicrobial evaluation was done in the Microanalytical Center at Cairo University.

### 3.1 General disc diffusion (agar-based) method

Standard discs of tetracycline (antibacterial agent) and amphotericin B (antifungal agent) served as positive controls and references for antimicrobial activities respectively, but filter discs impregnated with 10µL of solvent (chloroform, ethanol, DMF) were used as a negative control. The agar used is Mueller - Hinton agar that is rigorously tested for composition and pH. The depth of the agar in the plate is a factor to be considered in this method. Blank paper discs (Schleicher and Schuell, Spain) with a diameter of 8.0 mm were impregnated with 10 µL of the tested concentration of the stock solutions. When a filter paper disc impregnated with a tested chemical is placed on agar, the chemical will diffuse from the disc into the agar. This diffusion will place the chemical in the agar only around the disc. The solubility of the chemical and its molecular size will determine the size of the area of chemical infiltration around the disc. If an organism is placed on the agar it will not grow in the area susceptible to the chemical around the disc. This area of no growth around the disc is the “zone of inhibition” or “clear zone”. For disc diffusion, the zone diameters were measured with slipping calipers of the National Committee for Clinical Laboratory Standards (NCCLS) [20]. Agar-based method is a good alternative method being simpler and faster than broth–based methods [21, 22].

### 3.2 Antibacterial Activity

Concentration of 1 mg/mL of test compounds were prepared by dissolving the compounds in its proper solvent. For each concentration, 0.2 mL of synthesized compounds (1 mg/mL) was added to each hole. The plates were allowed to stand at room temperature for two hours and then incubated. The organisms were grown in nutrient agar at 37°C for 24 hours. After incubation period, the growth inhibition zones diameters were carefully measured in mm. The clear zone around the wells was measured as inhibition zones. The absence of a clear zone around the well was taken as inactivity.

Results of antibacterial activity tested against *E. Coli* (G-) and *S. Aureus* (G+) showed that all of the selected compounds were antibacterially active and comparatively efficient.

### 3.3 Antifungal Activity

The samples were dissolved, each in its proper solvent, then 0.5 mL sample of each compound (1 mg/mL) plus 0.1 mL of the tested fungal suspension were mixed thoroughly with 20 mL of agar medium, which was maintained at 45°C. The inoculated medium was poured into sterile Petri-dishes, allowed to solidify, and incubated at 25°C for seven days. Results of antifungal activity tested showed that compounds 4c, 4d, 6, 9 and 10 were active against both fungi, none was active with *A. flavus*, 4f, 4g, 8 and 15 were active only with *C. albicans*, whereas the rest of compounds were totally inactive.

All the results for the antimicrobial evaluation are given in (Table 1) showing the inhibition zone diameter in mm/mg sample. Both compounds 9 and 10 showed the highest inhibition with *S. aureus* whereas compounds 6 and 9 showed the highest inhibition towards *C. albicans*.

**Table 1:** *In vivo* antimicrobial activity by agar diffusion method of tested compounds

Compound	Inhibition zone diameter (mm / mg sample)				

	*E.* *coli*	*S. aureus*	*A.* *flavus*	*C.* *albicans*	Control solvent
Tetracycline	33	31	00	00	----
Amphotericin B	00	00	17	21	----
**4b**	10	10	00	00	Chloroform
**4c**	09	10	09	10	DMF
**4d**	16	16	12	15	Ethanol
**4f**	14	16	00	12	Ethanol
**4g**	14	15	00	12	Ethanol
**6**	18	18	17	16	Ethanol
**7**	08	08	00	00	Chloroform
**8**	12	12	00	12	Ethanol
**9**	08	21	08	18	Ethanol
**10**	08	21	08	12	Ethanol
**15**	14	16	00	16	Ethanol

In conclusion all the products 4b-d,f,g, 6-10 and 15 were antibacterially active and comparatively efficient. In addition, compounds 4c, 4d, 6, 9 and 10 were active against both fungi, 4f, 4g, 8 and 15 were active only with *C. albicans*, and the rest were inactive.

**Figure 1:**
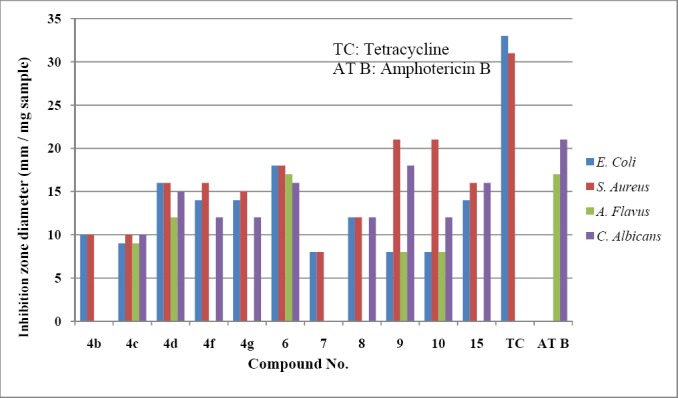
Graphical representation for the antimicrobial activity of the tested compounds

The antimicrobial activity of the products compared to those of tetracycline and amphotericin B are given in Fig 1.

## 4. Experimental

All melting points recorded are uncorrected. The IR spectra were recorded on a Pye Unicam SP1200 spectrophotometer using KBr wafer technique. The ^1^H-NMR spectra were determined on a Varian FT-200, Brucker AC-200 MHz instrument using TMS as an internal standard. Chemical shifts (δ) are expressed in ppm. The mass spectra were determined by EI technique using MP model NS-5988 and Shimadzu single focusing mass spectrometer (70 eV).

*2-Ethoxy-4(3H)quinazolinone* (1): 2-ethoxy(4*H*)-3,1-benzoxazin-4-one (0.01 mol) and ammonium acetate (0.01 mol) were fused using an oil bath for 2 h. The mixture was poured into an ice/water mixture and stirred. The off-white precipitate that separated out was filtered, washed, air-dried and crystallized from ethanol to give off-white crystals of compound 1. M.p. 155-156ºC; yield 85%; Anal. for C_10_H_10_N_2_O_2_ (m.w. 190); Found: C, 63.16; H, 5.26; N, 14.74; Calcd: C, 63.22; H, 5.18; N, 14.72; IR υ (cm^-1^) 1671 (C=O), 3229 (NH); MS: *m/z* (int. %) [M+] 190 (58%); H NMR (DMSO-d_6_) δ 1.19 (t, 3H; OCH_2_*CH_3_*, *J* = 7.4 Hz), 4.29 (q, 2H; -O*CH_2_*CH_3_, *J* = 7.4 Hz), 7.31-8.17 (m, 4H; ArH), 12.30 (br s, 1H, NH).

*General procedure for the synthesis of compounds* 2a-d and 3.

A mixture of quinazolinone 1 (0.01 mol) and any of the alkyl halides methyl iodide, ethyl iodide, benzyl chloride, ethyl chloroacetate or allyl bromide (0.01 mol) in dry acetone and anhydrous K_2_CO_3_ (50 mL/2 g) was heated under reflux for about 24 h. The excess acetone was distilled off and the residue was poured into cold water with stirring. The solid that separated out was filtered by suction, washed with water, dried and crystallized from suitable solvent affording derivatives 2–3, respectively.

*2-Ethoxy-3-methyl-4(3H)quinazolinone* (2a):

Light brown crystals from ethanol; m.p. 203-204 ºC; yield 70 %. Anal. for C_11_H_12_N_2_O_2_ (m.w. 204); Found: C, 64.82; H, 5.78; N, 13.68; Calcd: C, 64.70; H, 5.88; N, 13.72; IR υ (cm^-1^) 1686 (C=O), 2982 (CH); MS: *m/z* (int. %) [M+^•^] 204 (38.3); H NMR (DMSO-d_6_) δ 1.15 (t, 3H; -OCH_2_*CH_3_*, *J* = 7.4 Hz), 3.44 (s, 3H, N*CH_3_*), 4.29 (q, 2H; -O*CH_2_*CH_3_, *J* = 7.4 Hz), 7.36 - 7.97 (m, 4H; ArH).

*2-Ethoxy-3-ethyl-4(3H)quinazolinone* (2b):

Light brown crystals from ethanol; m.p. 217-218 ºC; yield 75 %. Anal. for C_12_H_14_N_2_O_2_ (m.w. 218); Found: C, 66.12; H, 6.38; N, 12.88; Calcd: C, 66.06; H, 6.42; N, 12.84; IR υ (cm^-1^) 1691 (C=O), 2982 (CH); MS: *m/z* (int. %) [M+^•^] 218 (47.3); H NMR (DMSO-d_6_) δ 1.19 (t, 3H; OCH_2_*CH_3_*, *J* = 7.4 Hz), 1.22 (t, 3H, NCH_2_*CH_3_*), 4.01 (q, 2H, N*CH_2_*CH_3_), 4.32 (q, 2H; -O*CH_2_*CH_3_, *J* = 7.4 Hz), 7.42 - 8.16 (4 d, 4H; ArH).

*3-Benzyl-2-ethoxy-4(3H)quinazolinone* (2c):

Light brown crystals from ethanol; m.p. 279-280 ºC; yield 85 %. Anal. for C_17_H_16_N_2_O_2_ (m.w. 280); Found: C, 72.82; H, 5.66; N, 10.14; Calcd: C, 72.86; H, 5.71; N, 10.00; IR υ (cm^-1^) 1689 (C=O), 2984 (CH); MS: *m/z* (int. %) [M+^•^] 280 (38.2); H NMR (DMSO- d_6_) δ 1.19 (t, 3H; OCH_2_*CH_3_*, *J* = 7.4 Hz), 4.35 (q, 2H; -O*CH_2_*CH_3_, *J* = 7.4Hz), 5.09 (s, 2H, *CH_2_*Ph), 7.25-7.33 (m, 5H, PhH), 7.38-8.17 (m, 4H; quinazolinone).

*2-Ethoxy-3-ethoxycarbonylmethyl-4(3H)quinazolinone* (2d):

Off-white crystals from ethanol; m.p. 275-276 ºC; yield 65 %. Anal. for C_14_H_16_N_2_O_4_ (m.w. 276); Found: C, 60.82; H, 5.83; N, 10.22; Calcd: C, 60.87; H, 5.80; N, 10.14; IR υ (cm^-1^) 1672, 1734 (2C=O), 2904 (CH); MS: *m/z* (int. %) [M+^•^] 276 (55.2); H NMR (DMSO-d_6_) δ 1.19 (t, 3H; OCH_2_*CH_3_*, *J* =7.4 Hz), 1.15 (t, 3H, COOCH_2_*CH_3_*), 4.96 (s, 2H, *CH_2_*CO), 4.24(q, 2H, COO*CH_2_*CH_3_), 4.34(q, 2H; O*CH_2_*CH_3_, *J* = 7.4Hz), 7.43-8.17(m, 4H; ArH).

*3-Allyl-2-ethoxy-4(3H)quinazolinone* (3):

White crystals from ethanol; m.p. 231-232 ºC; yield 75 %. Anal. for C_13_H_14_N_2_O_2_ (m.w. 230); Found: C, 67.80; H, 6.02; N, 12.22; Calcd: C, 67.83; H, 6.08; N, 12.17; IR υ (cm^-1^) 1619 (C=N), 2846, 2885 (CH), 1264 (C-O-C); MS: *m/z* (int. %) [M+] 230 (33.8); H NMR (DMSO-d_6_) δ 1.20 (t, 3H; -OCH_2_*CH_3_*, *J* = 7.4Hz), 2.46 (t, 2H, *J* = 6.4), 4.16 (q,2H; -O*CH_2_*CH_3_, *J* = 7.4 Hz), 4.73 (d, 2H, -*CH_2_* - CH=CH_2_), 5.01, 5.05 (2 dd, 2H, -CH_2_-CH=*CH_2_*), 6.01 (m, 1H, -CH_2_ - *CH*=CH_2_), 7.43 - 8.82 (m, 4H; ArH).

*General procedure for the synthesis of compounds* 4a-d and 5a-c.

To a solution of compound 1 (0.01 mol) in 50 mL of dry acetone was added the acid chloride, acetyl, *n*-decanoyl, phthalimidoacetyl, naphthalene sulphonyl, crotonyl, furoyl or benzoyl chlorides (0.04 mol) and anhydrous K_2_CO_3_ (0.04 mol). The reaction mixture was refluxed for 24 h. The excess acetone was evaporated and the residue was poured into cold water with stirring. The separated solid was filtered off, washed with cold water, dried and crystallized from the proper solvent to give 4 and 5, respectively.

*General procedure for the synthesis of compounds* 4a-g and 5a-g.

The quinazolinone 1 (0.01 mol) and the same acid chloride (0.04 mol) were fused at150 ºC in an oil bath for 2 h, and poured into an ice/water mixture. The solid that separated out was filtered, washed, dried, and then crystallized from the proper solvent affording 4a-g and 5a-g.

*Another procedure for the synthesis of* *derivatives* 4a-g.

A mixture of quinazolinone 1 (0.01 mol) and the acid chloride (0.01 mol) was refluxed in 50 mL of dry pyridine for 4 h. The excess solvent was distilled off and the reaction solution was cooled then poured into crushed ice with stirring leaving a crude product which was filtered off, washed with cold water, dried and crystallized from the proper solvent to afford 4a-g.

*3-Acetyl-2- ethoxy-4(3H)quinazolinone* (4a):

Brown crystals from ethanol; m.p. 221-222 ºC; yield 65%. Anal. for C_12_H_12_N_2_O_3_ (m.w. 232); Found: C, 62.09; H, 7.72; N, 12.05; Calcd: C, 62.07; H, 7.76; N, 12.07; IR υ (cm^-1^) 1671, 1734 (2xC=O), 2982 (CH); MS: *m/z* (int.%) [M+^•^] 232 (34.6); H NMR (DMSO-d_6_) δ 1.19 (t, 3H; -OCH_2_*CH_3_*, *J* = 7.4 Hz), 2.21 (s, 3H, -CO*CH_3_*), 4.36 (q, 2H; -O*CH_2_*CH_3_, *J* = 7.4 Hz), 7.44 - 8.20 (m, 4H, ArH).

*3-n-Decanoyl-2-ethoxy-4(3H)quinazolinone* (4b):

Yellow crystals from light petroleum (80-100 ºC); m.p. 145-146ºC; yield 75%. Anal. for C_20_H_28_N_2_O_3_ (m.w. 344); Found: C, 69.74; H, 8.18; N, 8.12; Calcd: C, 69.77; H, 8.14; N, 8.14; IR υ (cm^-1^) 1691, 1738 (C=O), 2925 (CH); MS: *m/z* (int. %) [M+^•^] 344 (56.7); H NMR (DMSO-d_6_) δ 0.87 (t, 3H, *CH_3_*), 1.23- 1.56 (m, 14H, *CH_2_* grps), 1.2 (t, 3H; OCH_2_*CH_3_*, *J* = 7.4Hz), 2.63(t, 2H, *CH_2_*CO), 4.36 (q, 2H; O*CH_2_*CH_3_, *J* = 7.4), 7.11-8.30 (m, 4H, ArH).

*2-Ethoxy-3-phthalamidoacetyl-4(3H)quinazolinone* (4c):

Yellow crystals from DMF; m.p. 297-298 ºC; yield 85%. Anal. for C_20_H_15_N_3_O_5_ (m.w. 377); Found: C, 63.62; H, 3.92; N, 11.18; Calcd: C, 63.66; H, 3.98; N, 11.14; IR υ (cm^-1^) 1667, 1714, 1774 (4xC=O), 2993 (CH); MS: *m/z* (int. %) [M+^•^] 377 (41.6); H NMR (DMSO-d_6_) δ 1.20 (t, 3H; -OCH_2_*CH_3_*, *J* = 7.1 Hz), 4.63 (m, 2H, *CH_2_*), 4.46 (q, 2H; -O*CH_2_*CH_3_, *J* = 7.1 Hz), 7.31-8.21 (m, 8H, ArH).

*2-Ethoxy-3-(1-naphthalenesulphonyl)-4(3H)quinazolinone* (4d):

Light brown crystals from ethanol; m.p. 182-183 ºC; yield 75 %. Anal. for C_20_H_16_N_2_O_4_S (m.w. 380); Found: C, 63.12; H, 4.19; N, 7.38; S, 8.42; Calcd: C, 63.16; H, 4.21; N, 7.38; S, 8.42; IR υ (cm^-1^) 1157 (S=O), 1671 (C=O); MS: *m/z* (int. %) [M+^•^] 380 (56.2); H NMR (DMSO-d_6_) δ 1.23 (t, 3H; -OCH_2_*CH_3_*, *J* = 6.8 Hz), 4.44 (q, 2H; -O*CH_2_*CH_3_, *J* = 6.8 Hz), 7.40-8.22 (m, 11H, ArH).

*3-Crotonyl-2-ethoxyquinazolin-4-one* (4e):

Off-white crystals from ethanol; m.p. 227-228 ºC; yield 75%. Anal. for C_14_H_14_N_2_O_3_ (m.w. 258); found: C, 65.18; H, 5.48; N, 10.81; Calcd: C, 65.11; H, 5.43; N, 10.85; IR υ (cm^-1^) 1671, 1728 (2 C=O), 2938 (CH); MS: *m/z* (int. %) [M+^•^] 258 (55.4); H NMR (DMSO-d_6_) δ 1.2 (t, 3H; -OCH_2_*CH_3_*, *J* = 7.4Hz), 1.86 (t, 3H, *CH_3_*), 4.36 (q, 2H; -O*CH_2_*CH_3_, *J* = 7.4 Hz), 6.35 (d, H, CH_trans_), 7.05 (d, H, CH_trans_), 7.39-8.2 (m, 4H, ArH).

*2-Ethoxy- 3-furan-2-oyl-quinazolin-4-one* (4f):

White crystals from ethanol; m.p.177-178 ºC; yield 80 %. Anal. for C_15_H_12_N_2_O_4_ (m.w. 284); found:C, 63.18; H, 5.48; N, 10.81; Calcd: C, 63.38; H, 4.23; N, 9.86; IR υ (cm^-1^) 1671, 1734 (2C=O); MS: *m/z* (int. %) [M+] 284 (56.1); H NMR (DMSO-d_6_) δ1.19 (t, 3H, -OCH_2_*CH_3_*, *J* = 7.4 Hz), 4.37 (q, 2H; -O*CH_2_*CH_3_, *J* = 7.4 Hz), 6.75 (dd, 1H, Furan-H), 7.34 (d, 1H, Furan-H), 7.91 (d, 1H, Furan-H), 7.46-8.20 (m, 4H, ArH).

*3-Benzoyl-2-ethoxyquinazolin-4-one* (4g):

White crystals from ethanol; m.p. 117-118 ºC; yield 65%. Anal. for C_17_H_14_N_2_O_3_ (m.w. 294); Found: C, 69.35; H, 4.73; N, 9.54; Calcd: C, 69.39; H, 4.76; N, 9.52; IR υ (cm^-1^) 1671, 1734 (2xC=O); MS: *m/z* (int. %) [M+] 294 (58.4); H NMR (DMSO-d_6_) δ 1.2 (t, 3H, -OCH_2_*CH_3_*, *J* = 7.4 Hz), 4.37 (q, 2H; -O*CH_2_*CH_3_, *J* = 7.4 Hz), 7.46-8.2 (m, 9H, ArH).

*[2-Ethoxyquinazolin-4-yl] acetate* (5a):

Off-white crystals from ethanol; m.p. 167-168 ºC; yield 65%. Anal. for C_12_H_12_N_2_O_3_ (m.w. 232); Found: C, 62.13; H, 7.70; N, 12.10; Calcd: C, 62.07; H, 7.76; N, 12.07; IR υ (cm^-1^) 1605 (C=N), 1780 (C=O); MS: *m/z* (int. %) [M+] 232 (67.5); H NMR (DMSO-d_6_) δ 1.2 (t, 3H, -OCH_2_*CH_3_*, *J* = 7.4 Hz), 2.14 (s, 3H, -CO*CH_3_*), 4.19 (q, 2H; -O*CH_2_*CH_3_, *J* = 7.4 Hz), 7.50-8.85 (m, 4H, ArH).

*[2-Ethoxyquinazolin-4-yl]-n-decanoate* (5b):

Light brown crystals from benzene; m.p. 167-168ºC; yield 70%. Anal. for C_20_H_28_N_2_O_3_ (m.w. 344); Found: C, 69.79; H, 8.18; N, 8.14; Calcd: C, 69.77; H, 8.14; N, 8.14; IR υ (cm^-1^) 1619 (C=N), 1769 (C=O); MS: *m/z* (int. %) [M+] 344 (44.8); H NMR (DMSO-d_6_) δ 0.87 (t, 3H, *CH_3_*), 1.23-1.56 (m, 14H, *CH_2_* grps), 1.2 (t, 3H, OCH_2_*CH_3_*, *J* = 7.4 Hz), 2.4 (t, 2H, *CH_2_*CO), 4.19 (q, 2H; -O*CH_2_*CH_3_, *J* = 7.4 Hz), 7.51-8.85 (m, 4H, ArH).

*[2-Ethoxyquinazolin-4-yl]phthaloylglycinate* (5c):

Off-white crystals from ethanol; m.p. 272-273ºC; yield 65%. Anal. for C_20_H_15_N_3_O_5_ (m.w. 377); Found: C, 63.68; H, 3.94; N, 11.16; Calcd: C, 63.66; H, 3.98; N, 11.14; IR υ (cm^-1^) 1590 (C=N), 1730, 1774 (3 x C=O), 2990 (CH); MS: *m/z* (int. %) [M+] 377 (66.5); H NMR (DMSO-d_6_) δ 1.21 (t, 3H, -OCH_2_*CH_3_*, *J* = 7.4 Hz), 4.7 (m, 2H, -*CH_2_*), 4.17 (q, 2H; -O*CH_2_*CH_3_, *J* = 7.4 Hz), 7.69-8.85 (m, 8H, Ar-H).

*[2-Ethoxyquinazolin-4-yl]naphthalene-1-sulphonate* (5d):

Brown crystals from light petroleum (80-100 ºC); m.p. 152-153 ºC; yield 70 %. Anal. for C_20_H_16_N_2_O_4_S (m.w. 380); Found: C, 63.18; H, 4.16; N, 7.40; S, 8.44; Calcd: C, 63.16; H, 4.21; N,7.38; S, 8.42; IR υ (cm^-1^) 1163 (S=O), 1620 (C=N); MS: *m/z* (int. %) [M+] 380 (72.3); H NMR (DMSO-d_6_) δ 1.21 (t, 3H, OCH_2_*CH_3_*, *J* = 6.8 Hz), 4.15 (q, 2H; -O*CH_2_*CH_3_, *J* = 6.8 Hz), 7.68-8.86 (m, 11H, ArH).

*[2-Ethoxyquinazolin-4-yl] crotonate* (5e):

Light brown crystals from benzene; m.p. 197-198 ºC; yield 65 %. Anal. for C_14_H_14_N_2_O_3_ (m.w. 258); Found: C, 65.15; H, 5.42; N, 10.79; Calcd: C, 65.11; H, 5.43; N, 10.85; IR υ (cm^-1^) 1264 (C-O-C), 1617 (C=N), 1767 (C=O), 2881, 2939 (CH); MS: *m/z* (int. %) [M+] 258 (68.4); H NMR (DMSO-d_6_) δ 1.2 (t, 3H, -OCH_2_*CH_3_*, *J* = 4.7 Hz), 1.89 (t, 3H, -*CH_3_*), 4.19 (q, 2H; O*CH_2_*CH_3_, *J* = 4.7 Hz), 6.19 (d, H, CH_trans_), 7.24 (d, H, CH_trans_), 7.50-8.85 (m, 4H, ArH).

*[2-Ethoxyquinazolin-4-yl] furan-2-carboxylate* (5f):

Off-white crystals from benzene; m.p. 153-154ºC; yield 75%. Anal. for C_15_H_12_N_2_O_4_ (m.w. 284); Found: C, 65.18; H, 5.48; N, 10.81; Calcd: C, 63.38; H, 4.23; N, 9.86; IR υ (cm^-1^) 1588 (C=N), 1763 (C=O); MS: *m/z* (int. %) [M+] 284 (44.3); H NMR (DMSO-d_6_) δ 1.2 (t, 3H, -OCH_2_*CH_3_*, *J* = 7.4 Hz), 4.17 (q, 2H; -O*CH_2_*CH_3_, *J* = 7.4 Hz), 6.80 (dd, 1H, Furan-H), 7.32 (d, 1H, Furan-H), 7.98 (d, 1H, Furan-H), 7.61-8.85 (m, 4H, ArH).

*[2-Ethoxyquinazolin-4-yl] benzoate* (5g):

Off-white crystals from benzene; m.p. 101-102 ºC; yield 70 %. Anal. for C_17_H_14_N_2_O_3_ (m.w. 294); Found: C, 69.35; H, 4.73; N, 9.54; Calcd: C, 69.39; H, 4.76; N, 9.52; IR υ (cm^-1^) 1611 (C=N), 1769 (C=O); MS: *m/z* (int. %) [M+^•^] 294 (55.8); H NMR (DMSO-d_6_) δ 1.2 (t, 3H, OCH_2_*CH_3_*, *J* = 7.4 Hz), 4.17 (q, 2H; O*CH_2_*CH_3_, *J* = 7.4 Hz), 7.55-8.85 (m, 9H, ArH).

*General procedure for the synthesis of compounds* 6, 7, and 8.

Compound 1 (0.01 mol) was refluxed with reagents such as p-acetylaminophenylsulphonyl chloride, ethyl chloroformate and chloroacetyl chloride (0.01 mol) in 50 mL of dry pyridine for 4 h. The excess solvent was distilled off and the solution was left to cool and then poured onto ice with stirring to obtain the crude product which was filtered off, thoroughly washed with cold water, dried and crystallized from the proper solvent affording products 6, 7 and 8 respectively.

*2-Ethoxy-3-(p-acetylaminophenylsulphonyl) quinazolin-4-one* (6):

White crystals from ethanol; m.p. 261-262ºC; yield 88 %. Anal. for C_18_H_14_N_3_O_5_S (m.w. 387); Found: C, 55.79; H, 4.41; N, 10.89; S, 8.31; Calcd: C, 55.81; H, 4.39; N, 10.85; S, 8.27; IR υ (cm^-1^) 1160 (S=O), 1666, 1712 (2 C=O), 2992 (CH), 3271 (NH); MS: *m/z* (int. %) [M+] 387 (68.2); H NMR (DMSO-d_6_) δ 1.23 (t, 3H, -OCH_2_*CH_3_*, *J* = 7.4 Hz), 2.13 (s, 3H, CO*CH_3_*), 4.44 (q, 2H; -O*CH_2_*CH_3_, *J* = 7.4 Hz), 7.72 (d, 1H, NH, *J* = 7.1 Hz), 7.45-8.18 (m, 8H; quinazolinone-H and ArH). ^13^C-NMR: 15.0, 64.6, 154.0, 146.9, 120.8, 161.9, 126.7, 133.4, 127.3, 126.6, 129.4, 132.2, 129.4, 118.0, 144.8, 118.0, 168.9, 24.0.

*3-Ethoxycarbonyl-2-ethoxyquinazolin-4-one* (7):

White crystals from benzene; m.p. 177-178ºC; yield 92%. Anal. for C_13_H_14_N_2_O_4_ (m.w. 262); Found: C, 59.65; H, 5.39; N, 10.68; Calcd: C, 59.54; H, 5.34; N, 10.69; IR υ (cm^-1^) 1672, 1762 (2 C=O); MS: *m/z* (int. %) [M+] 262 (88.5); H NMR (DMSO-d_6_) δ 1.19 (t, 3H, -OCH_2_*CH_3_*, *J* = 7.4 Hz), 1.15 (t, 3H, CH_3_, COOCH_2_*CH_3_*), 4.24 (q, 2H, CH_2_, COO*CH_2_*CH_3_), 4.34 (q, 2H; -O*CH_2_*CH_3_, *J* = 7.4 Hz), 7.43-8.17 (m, 4H, ArH).

*3-Chloroacetyl-2-ethoxyquinazolin-4-one* (8):

Brownish white crystals from ethanol; m.p. 152-153ºC; yield 85%. Anal. for C12H11N2O3Cl (m.w. 266.5); Found: C, 54.08; H, 4.15; N, 10.56; Cl, 13.32; Calcd: C, 54.03; H, 4.13; N, 10.51; Cl, 13.28; IR υ (cm-1)1668, 1717 (2 x C=O), 2823 (CH); MS: m/z (int. %) [M+] 266.5 (77.3); H NMR (DMSO-d6) δ 1.2 (t, 3H, -OCH2CH3, J = 7.4 Hz), 4.36 (q, 2H; -OCH2CH3, J = 7.4 Hz), 4.29 (d, 2H, CH2Cl), 7.45-8.20 (m, 4H, ArH).

*Synthesis of Compounds* 9 *(acetylated α and β anomers) and* 10 *(deacetylated α and β anomers)*

A crude mixture of compound 1 (0.05 mol) and α-bromoglucose tetraacetate (0.01 mol) in 1,4-dioxane (100 mL) was heated with stirring under reflux for 4 h. The mixture was cooled to room temperature and the solvent was removed under reduced pressure. The residue was then dissolved in ethyl acetate, washed sequentially with saturated NaHCO3, dried over MgSO4. Purification and separation was achieved using column chromatography (3:1 EtOAc: Hexane) affording the acetylated derivative 9 as a white solid which was later on crystallized using dichloromethane-diethyl ether -hexane solvents. A solution of product 9 in MeOH (0.004 mol/65mL) was treated with sodium carbonate solution (0.002 mol). A white syrupy solid began to precipitate. The residue was purified using column chromatography (3:1, EtOAc:Hexane) to give the deacetylated product as a white solid, which was later on crystallized using dichloromethane-diethyl ether-hexane solvents to give the crystalline product 10 (α and β anomers).

*N-(2, 3, 4, 6-Tetra-0-acetylglucopyranosyl)quinazolin-4-one (α + β)* (9):

*α-Anomer*: Compound was obtained as amorphous white crystalline solid from methanol; yield 14 %; m.p. 182-183°C. Anal. for C_24_H_28_N_2_O_11_ (m.w. 520); Found: C, 55.22; H, 5.41; N, 5.48; Calcd: C, 55.38; H, 5.38; N, 5.38; IR υ (cm^-1^) 1662, 1738 (2 C=O); MS: *m/z* (int. %) [M+] 520 (33.6); H NMR (CDC1_3_) δ 1.2 (t, 3H, -OCH_2_*CH_3_*, *J* = 7.4 Hz), 2.05-2.06 (s, 12H, 4 Ac-H), 3.74-5.12 (m, 6H, H-2, H-3, H-4, H-5, H-6_a_, H-6_b_), 4.45 (q, 2H; -O*CH_2_*CH_3_, *J* = 7.4 Hz), 6.17 (d, 1H, H_anom_), 7.40-7.85 (m, 4H, ArH).

β-Anomer: Position of the spot on TLC plate was lower to the α-anomer, flaky amorphous solid; yield 17 %; m.p. 169-172 °C. Anal. for C24H28N2O11 (m.w. 520); Found: C, 55.48; H, 5.31; N, 5.24;Calcd: C, 55.38; H, 5.38; N, 5.38; IR υ (cm-1) 1666, 1738 (2 x C=O); MS: m/z (int. %) [M+] 520 (38.3); H NMR (CDC13) δ 1.20 (t, 3H, -OCH2CH3, J = 7.4 Hz), 2.05-2.06 (s, 12H, Ac-H), 3.74-5.11 (m, 6H, H-2, H-3, H-4, H-5, H-6a, H-6b), 4.45 (q, 2H; -OCH2CH3, J = 7.4 Hz), 5.81 (d, 1H, Hanom), 7.28-7.85 (m, 4H, ArH).

*2-Ethoxy-3-(glucopyranosyl-2-yl)quinazolin-4-one* (10) (α-anomer):

White syrupy crystals of α-anomer from methanol; m.p. 197-198 ºC; yield 24%. Anal. for C_16_H_20_N_2_O_7_ (m.w. 352); Found: C, 54.52; H, 5.69; N, 7.92; Calcd: C, 54.54; H, 5.68; N, 7.95; IR υ (cm^-1^) 1662 (C=O), 3268 (OH bonded), 3384 (OH non-bonded); MS: *m/z* (int. %) [M+] 352 (33.8); H NMR (D_2_O) δ 1.2 (t, 3H, OCH_2_*CH_3_*, *J* = 6.8 Hz), 3.17-3.87 (m, 5H, H-2, H-3, H-4, H-5, H-6_a_, H-6_b_), 3.58 (m, 2’-OH, 3’-OH, 4’-OH), 3.65 (s, 6’-OH), 6.09 (d, 1H, H-1), 4.34 (q, 2H; -O*CH_2_*CH_3_, *J* = 6.8 Hz), 7.29-8.18 (m, 4H, ArH).

*2-Ethoxy-3-(glucopyranosyl-2-yl)quinazolin-4-one* (10) (β-anomer):

Amorphous flaky solid of β-anomer from 1,4-dioxane; m.p. 189-191ºC; yield 14 %. Anal. for C16H20N2O7 (m.w. 352); Found: C, 54.52; H, 5.69; N, 7.92; Calcd: C, 54.54; H, 5.68; N, 7.95; IR υ (cm-1) 1668 (C=O), 3247 (OH bonded), 3362 (OH non-bonded); MS: m/z (int. %) [M+•] 352 (36.2); H NMR (D2O) δ 1.1 (t, 3H, OCH2CH3, J = 6.8 Hz), 3.54-4.79 (m, 5H, H-2, H-3, H-4, H-5, H-6a, H-6b), 3.58 (m, 2’-OH, 3’-OH, 4’-OH), 3.65 (s, 6’-OH), 5.78 (d, 1H, H-1), 3.58 (q, 2H; -OCH2CH3, J = 6.8 Hz), 7.63-8.03 (m, 4H, ArH).

*4-Chloro-2-ethoxyquinazoline* (11):

A solution of 2-ethoxy-4(3H)quinazolinone 1 (0.01 mol) with phosphorus oxychloride (20 mL) was heated on a water bath at 70°C for 2 h. The reaction mixture was cooled and diluted with ice-water and the resulting precipitate was collected by filtration and then crystallized from chloroform affording product 11. Light brown crystals from ethanol; m.p. 180-182 ºC; yield 85 %. Anal. for C10H9N2OCl (m.w. 208.5); Found: C, 57.45; H, 4.31; N, 13.42; Cl, 17.00; Calcd: C, 57.55; H, 4.30; N, 13.43; Cl, 17.02; IR υ (cm-1) 1622 (C=N); MS: m/z (int. %) [M+] 208.5 (57.9); H NMR (DMSO-d6) δ 1.19 (t, 3H, -OCH2CH3, J = 7.4 Hz), 4.19 (q, 2H; -OCH2CH3, J = 7.4 Hz), 7.49-8.86 (m, 4H, ArH).

*2-Ethoxy-4(3H)quinazolin-4-thione* (12):

A solution of quinazolinone 1 and P2S5 (0.03 mol each) in dry xylene (50 mL) was boiled for 6 h. The reaction mixture was filtered while hot and then concentrated. The solid separated on cooling was crystallized from the suitable solvent to give product 12 as brown crystals from ethanol; m.p. 137-138 ºC; yield 65 %. Anal. for C10H10N2OS (m.w. 206); Found: C, 58.15; H, 4.81; N, 13.52; S, 15.53; Calcd: C, 58.25; H, 4.85; N, 13.59; S, 15,53; IR υ (cm-1) 1319 (C=S), 1597 (C=N), 3137 (NH); MS: m/z (int. %) [M+•] 206 (55.7); H NMR (DMSO-d6) δ 1.19 (t, 3H, OCH2CH3, J = 7.4Hz), 4.39 (q, 2H; OCH2CH3, J = 7.4), 7.29-7.67 (m, 4H, ArH), 12.3 (br s, 1H, NH).

*3-[2-Ethoxyquinazolin-3-yl]propionitrile* (13):

A mixture of quinazolinone 1 and acrylonitrile (0.01 mol each) was heated under reflux in DMF (30 mL) for 5h. The reaction mixture was then poured into crushed ice. The solid that precipitated out was filtered, washed dried and crystallized from ethanol to give white crystals of product 13; m.p. 190-192 ºC; yield 85 %. Anal. for C13H13 N3O2 (m.w. 243); Found: C, 64.14; H, 5.29; N, 17.32; Calcd: C, 64.20; H, 5.35; N, 17.28; IR υ (cm-1) 1675 (C=O), 2246 (CN), 2989 (CH); MS: m/z (int. %) [M+•] 243 (69.3); H NMR (DMSO-d6) δ 1.19 (t, 3H, -OCH2CH3, J = 7.4Hz), 3.14 (t, 2H, CH2CN), 4.15 (t, 2H, NCH2), 4.2 (q, 2H;-OCH2CH3, 7.42-8.16 (m, 4H, ArH).

*2-(2-Ethoxyquinazolin-4-one-3-ylmethyl)-5-(2,3,4,6-tetraacetyl-β-glucopyranosyl-2-ylamino)thiadiazole* (14):

*Preparation of the reagent for compound* (14):

A mixture of thiosemicarbazide (0.01 mol) and chloroacetyl chloride (0.015 mol) was heated under reflux for 2h. The reaction mixture was then poured into crushed ice. The precipitated solid was filtered, washed, dried and crystallized from the proper solvent to afford 2-chloromethyl-5-aminothiadiazole. A mixture of this product and α-bromoglucose tetraacetate (0.01mol each) in anhydrous acetonitrile (300 mL) was left for 24 h at room temperature with occasional stirring. The suspension was filtered through Celite and the filtrate was taken to dryness. The resulting residue was purified by flash chromatography using a gradient of hexane/ethyl acetate (2:1 to 1:1) affording the acetylated α-anomer of the reagent, the 2-chloromethyl-5-N- (2,3,4,6-tetra-O- acetyl-β-glucopyranosyl) aminothiazole. A mixture of this reagent (0.01 mol) and quinazolinone 1 (5 eqv.) was heated under reflux in 1,4- dioxane (50 mL) for 8 h. The excess solvent was then evaporated and the residue was poured into cold water with stirring. The solid that separated out was filtered, washed with cold water, dried and crystallized from DMF to give light brown crystals of the acetylated product of derivative 14; m.p. above 300ºC. Deacetylation was achieved by adding CH3ONa solution (0.01mol) to this derivative (0.02mol) in methanol (65 mL). White crystals began to precipitate within 5 minutes by quenching with acetic acid (0.003 mmol). The solvent was removed in vacuo and the residue was purified; m.p. 133-135 ºC; yield 65 %. Anal. for C29H33N5O12S (m.w. 633); Found: C, 54.99; H, 5.18; N, 11.05; S, 5.04; Calcd: C, 54.97; H, 5.21; N, 11.06; S, 5.06; IR υ (cm-1) 1669 (C=O), 1768 (OAc), 2992 (CH), 3123 (NH); MS: m/z (int. %) [M+] 465 (33.8); H NMR (DMSO-d6) δ 1.44 (t, 3H, OCH2CH3, J = 6.8 Hz), 2.05-2.06 (4 s, 12H, 4OAc), 2.6-2.7 (br. s,1H, NH), 5.29 (s, 2H, NCH2), 3.88 (dt, 1H, H-3’), 4.49 (d, 1H, H-4’), 4.52 (q, 2H; -OCH2CH3, J = 6.8 Hz), 4.91 (2d, 2H, H-6a’ and H-6b’), 4.95 (t, 1H, H-5’), 5.02 (dd, 1H, H-2’), 5.47 (d, 1H, H-1’), 7.32-7.93 (m, 4H, ArH).

*N-Acetyl-2-(2-ethoxyquinazolin-4-one-3-ylmethyl)-5-(2,3,4,6-tetraacetyl-β-glucopyranosyl-2-ylamino)thiadiazole* (15):

A cold solution of product 14 (0.01 mol) in dry pyridine (25 mL) was reacted with Ac2O (25 mL). The mixture was kept overnight at room temperature, with occasional shaking, and then poured onto crushed ice, and the residue was collected by filtration, washed repeatedly with water, air-dried and crystallized from ethanol affording product 15; m.p. 123-125 ºC; yield 65 %. Anal. for C27H31N5O11S (m.w. 675); Found:C, 48.08; H, 4.69; N,10.54; S, 4.84; Calcd: C, 48.00; H, 4.59; N,10.37; S, 4.74; IR υ (cm-1) 1669, 1772 (2C=O), quinazolinone and OAc), 2992 (CH); MS: m/z (int. %) [M+•] 465 (66.6); H NMR (DMSO-d6) δ 1.44 (t, 3H, -OCH2CH3, J = 6.8 Hz), 2.06-2.15 (5 s, 15 H, 5 OAc), 5.27 (s, 2H, NCH2), 4.89 (dt, 1H, H-3’), 5.01 (d, 1H, H-4’), 4.53 (q, 2H; OCH2CH3, J = 6.8Hz), 4.49 (2d, 2H, H-6a’ and H-6b’), 3.77 (dt, 1H, H-5’), 5.18 (dd, 1H, H-2’), 5.93 (d, 1H, H-1’), 7.47-7.96 (m, 4H, ArH). 13C-NMR: 15.0, 64.9, 150.1, 126.7, 133.4, 127.3, 126.6, 146.9, 120.8, 161.6, 40.7, 168.0, 163.4, 21.0, 169.9, 23.3, 170.2, 95.7, 68.2, 81.9, 28.7, 51.3, 170.2, 20.4, 65.6, 62.4, 170.2, 21.0, 170.2, 20.7.

*1-(p-Methoxyphenyl)-3-(p-chlorophenyl)-3-(2-ethoxyquinazolin-4-one-3-yl) propan-1-one* (16):

A mixture of compound 1 and 4’-methoxyphenyl-4-chlorophenyl chalcone (0.01mol each) in ethanol (50 mL) was heated under reflux for 6 h. The solid that separated out was filtered, washed and air-dried. The residue was purified by chromatography using a gradient of hexane and ethyl acetate (2:1 to 1:1) to afford product 16; pale yellow crystals from methanol; m.p. 154-156 ºC; yield 75 %. Anal. for C26H23N2O4Cl (m.w. 462.5); Found: C, 67.48; H, 5.01; N, 6.01; Cl, 7.70; Calcd: C, 67.46; H, 4.97; N, 6.05; Cl, 7.68; IR υ (cm-1)1671, 1713 (2 C=O), 2990 (CH); MS: m/z (int. %) [M+] 462.5 (56.8); H NMR (DMSO-d6) δ 1.22 (t, 3H, OCH2CH3, J = 7.4 Hz), 2.72 (d, 2H, CH2CO), 3.8 (s, 3H, OCH3), 5.61 (t, 1H, -CH), 4.51 (q, 2H; -OCH2CH3, J = 7.4 Hz), 6.99-7.98 (m, 4H, -C6H4OCH3); 7.45-7.63 (m, 4H, -C6H4Cl); 7.45-8.17 (m, 4H, quinazolinone).

*2-Ethoxy-3-{2-hydroxy-3-oxo-1-(4-chlorophenyl)-3-(4-methoxyphenyl) quinazolin-4(3H)-one* (17):

Compound 1 (0.01mol) and trans-1-(4-methoxyphenyl)-3-(chlorophenyl)- 2-oxiranylpropanone (0.01 mol) in absolute ethanol (50mL) were heated together under reflux for 5 h. The solid that separated out was filtered, washed, dried and crystallized from methanol to afford pale yellow crystals of product 17. The purity of 17 was checked by chromatography and change in the melting point, 149-151 ºC, compared with that of reactant; yield 75%. Anal. for C26H23N2O5Cl (m.w. 478.5); Found: C, 65.23; H, 4.86; N, 5.82; Cl, 7.37; Calcd: C, 65.20; H, 4.80; N, 5.85; Cl, 7.41; IR υ (cm-1) 1671, 1719 (2C=O), 2966 (CH), 3223 (OH); MS: m/z (int. %) [M+] 478.5 (58.3); H NMR (DMSO-d6) δ 1.22 (t, 3H, -OCH2CH3, J = 7.4 Hz), 2.80 (1H, br s, OH), 4.82 (d, 1H, CHCO), 3.8 (s, 3H, OCH3), 5.52 (m, 1H, CH-C6H4 Cl), 4.44 (q, 2H; -OCH2CH3, J = 7.4 Hz), 7.06-8.01 (m, 4H, -C6H4OCH3); 7.46-7.53 (m, 4H, -C6H4Cl); 7.23-8.17 (m, 4H, quinazolinone). 13C-NMR: 15.0, 64.9, 150.1, 126.7, 133.4, 127.3, 126.6, 120.8, 146.9, 161.3, 52.5, 141.6, 127.2, 128.6, 132.3, 128.6, 127.2, 88.2, 197.0, 126.5, 129.8, 129.8, 114.2, 114.2, 165.0, 55.8.
